# Proteomic Insights into Heroin Use: Links to Neurodegeneration

**DOI:** 10.1007/s12035-026-05757-4

**Published:** 2026-02-21

**Authors:** Mustafa Gani Sürmen, Sadrettin Pence, Saime Sürmen, Yalcin Buyuk, Sibel Kuras, Birsen Elibol, Halime Hanim Pence

**Affiliations:** 1 Department of Molecular Medicine, Hamidiye Institute of Health Sciences, University of Health Sciences, Istanbul, Turkey; 2 Department of Physiology, Faculty of Medicine, Istanbul Medeniyet University, Istanbul, Turkey; 3 Department of Forensic Art, Institute of Forensic Sciences and Legal Medicine, Istanbul University-Cerrahpasa, Istanbul, Turkey; 4 Department of Medical Biochemistry, Hamidiye School of Medicine, University of Health Sciences Türkiye, Istanbul, Turkey; 5 Department of Medical Biology, Faculty of Medicine, Istanbul Medeniyet University, Istanbul, Turkey

**Keywords:** Heroin, Label-free quantification, Mass spectrometry, Neurodegeneration, Proteomics

## Abstract

Due to a lack of information related to molecular changes in heroin use, we aimed to examine heroin-dependent alterations in different regions of the post-mortem human brain. Tissues were obtained from males (n = 24 heroin users, n = 24 controls) through the Turkish Forensic Medicine Institute after structured verbal interviews with the relatives of the deceased to gather history of substance use. Following toxicological confirmation of heroin use, the hippocampus, putamen, and caudate nucleus were dissected from the left hemispheres. Proteomic analyses were performed using a high-resolution liquid chromatography-electrospray ionization-tandem mass spectrometry (LC–ESI–MS/MS) system. Label-free quantitative analysis revealed significant differential expression of 87 proteins in the hippocampus, 121 proteins in the putamen, and 80 proteins in the caudate nucleus compared to controls. These differentially expressed proteins (DEPs) were subsequently used to construct protein–protein interaction (PPI) networks using the STRING database, revealing significantly enriched and highly interconnected interaction networks in all three regions. Gene Ontology (GO) enrichment analysis of DEPs consistently identified extracellular exosome, extracellular space, and vesicle as the top three cellular components. Molecular function enrichment further indicated alterations in signaling, binding, and stress-related processes. The expression of TST, RYR2, ACTBL2, and RPS27 decreased, whereas the expression of COL4A2, OGN, PMP2, and MAP2K6 increased in the hippocampus. In the putamen, the most prominent increases were observed in DNM2 and MADD expression. In the caudate nucleus, the expressions of RPS6KA2, TMED10, and NBEA proteins decreased, whereas HPX protein expression increased. Overall, these alterations promote oxidative stress and molecular changes linked to neurodegeneration, which likely contribute to impaired neuronal function and synaptic plasticity.

**Supplementary Information:**

The online version contains supplementary material available at 10.1007/s12035-026-05757-4.

## Introduction

Heroin is a highly addictive opioid and exerts serious acute and chronic effects on neuronal and hormonal systems. In addition to the central nervous system, many tissues and organ systems throughout the body are severely affected by long-term heroin use [1]. The limbic system, which includes the hippocampus, prefrontal cortex, amygdala, dorsal striatum, ventral tegmental area, and nucleus accumbens, is among the most profoundly affected regions of the brain [2]. Post-mortem brain tissues obtained from heroin users provide critical information for elucidating heroin use-related morphological alterations in brain structure and composition, as well as the mechanisms underlying various cellular and molecular events [3, 4].

Thanks to various advances in science and improved methodologies, the former belief that substance abuse was a moral failing indicative of a lack of willpower has changed dramatically. In addition to traditional molecular and cellular research methods, modern -omics technologies have emerged as powerful tools for obtaining detailed information about changes in the brain due to heroin abuse, as well as other physiological and pathological conditions. Among these approaches, mass spectrometry-based proteomic approaches provide the opportunity to study proteins on a global and in-depth scale. To date, proteomic analyses have offered valuable insights into the proteomes of different brain regions in both humans and animals. For instance, many studies have used proteomics to discover biomarkers that may contribute to the diagnosis and treatment of neurodegenerative diseases [5, 6]. However, due to several limitations associated with studying human brain tissue such as its complex and heterogeneous structure and the invasive procedures required to obtain it, the number of studies investigating proteomic alterations in the human brain in relation to addictive substances remains limited [7]. These previous studies have shown that certain protein classes may be more sensitive to addictive drugs [8]. For example, proteomic analysis has detected morphine-related changes in metabolic, mitochondrial, synaptic, cytoskeletal, signal transduction, and oxidative stress-related proteins in rat brain tissue [9, 10]. Some evidence also indicates that heroin may contribute to hippocampal dysfunction, hyperphosphorylated tau accumulation, and impaired neurogenesis [4, 11]. Nevertheless, the development of heroin-related neurobiological disorders at the proteome level has not been fully elucidated. Furthermore, the effects of heroin on different brain regions in humans have not been examined in detail using proteomic methods.

While heroin-related changes have been extensively investigated through candidate protein and molecular studies, such approaches are inherently limited because they focus on predefined targets and therefore cannot capture the full complexity of molecular alterations induced by chronic opioid exposure [12, 13]. In contrast, proteomic approaches provide an unbiased, system-level perspective that enables the simultaneous evaluation of thousands of proteins, revealing novel molecular signatures and interconnected pathways that would otherwise remain undetected. A recent large-scale mass-spectrometry-based analysis [12] identified widespread changes in synaptic proteins associated with heroin use and specifically pointed out that the orbitofrontal cortex Shisa7 protein is a critical driver of neurobehavioral pathology related to drug-seeking behaviour. Similarly, an earlier study [14] reported alterations in cytoskeletal proteins, which function in the pathophysiology of drug addiction by disrupting neurotransmission and cellular signalling in post-mortem amygdala tissue from heroin users. These findings highlight the capacity of proteomics to uncover complex and multifactorial molecular disruptions beyond the reach of candidate-based methods. Therefore, adopting a proteomic strategy in the present study offers a significant advantage by enabling a comprehensive and unbiased characterization of heroin-associated alterations across different brain regions, thereby contributing novel insights into the neurobiological mechanisms underlying opioid addiction.

Accordingly, the present study aimed to reveal the proteome profiles of the hippocampus, putamen, and caudate nucleus from post-mortem tissue of heroin users in order to elucidate the molecular mechanisms underlying heroin’s effects on the brain and to provide a valuable resource for further studies. These regions were selected based on their well-established roles in addiction-related neurobiology and their vulnerability to chronic opioid exposure. The hippocampus is a key structure involved in learning, memory, and synaptic plasticity, functions that are significantly impaired in heroin addiction [15]. The caudate nucleus and putamen, as major components of the dorsal striatum, are critically involved in reward processing, habit formation, and motor control, all of which undergo maladaptive changes in substance use disorders [16]. Previous neuroimaging and post-mortem studies have reported structural and functional abnormalities in these regions in opioid-dependent individuals [17, 18]. Together, these regions were selected to provide a comprehensive understanding of how long-term heroin use alters neural circuits implicated in both cognitive dysfunction and addictive behaviours. Moreover, to further contextualize the proteomic findings, we focused on a subset of proteins previously linked to neurodegenerative processes, highlighting how their expression varies across regions associated with heroin exposure.

## Material and Methods

### Case Information

Post-mortem human brain tissues were obtained from the Turkish Ministry of Justice, the Institution of Forensic Medicine, with the necessary legal permissions (07.02.2014/B.03.1.ATK.0.01.00.08/111). Only male subjects were included in the present study. According to the toxicological analysis performed at the Institution of Forensic Medicine using a gas chromatography-flame ionization detector (GC-FID), individuals testing positive for 6-monoacetylmorphine (6-MAM), a specific metabolite of heroin, were initially identified. Furthermore, as part of standard forensic procedures in Turkey, the Office of the Public Prosecutor and the Forensic Medicine Council carried out formal interviews with the next of kin of the deceased to obtain information regarding any history of drug use. In this way, positive findings for 6-MAM were considered toxicological proof of recent use of heroin, while the documented history was considered evidence of chronic use. In the present study, only cases with previous reports of heroin use and testing positive for 6-MAM (in urine, blood, or hair samples) were considered heroin users. According to the available information, individuals who underwent autopsy due to heroin overdose were found to have occasionally used other substances, including morphine, codeine, amphetamines, benzodiazepines, and cocaine. Age-matched male controls were selected among deceased individuals whose autopsies were conducted at the Forensic Medicine Institution during the same period and who had no history or toxicological evidence of drug use. Causes of death in the control group included various cardiac pathologies, a vaccine-associated fatality, internal haemorrhage secondary to traumatic injury, drowning, and motor vehicle accidents. In the heroin user group, the post-mortem interval (PMI) averaged 22.6 ± 6.4 h, ranging from 5 to 43 h, whereas in the control group, the PMI averaged 20.8 ± 8.1 h with a range of 5 to 40 h. Individuals whose cause of death was brain tumours, epilepsy, acute infection and head trauma were excluded from the study.

The left hemispheres of both the heroin user group (n = 24, mean age = 31.5 ± 9.7 years) and the control group (n = 24, mean age = 33.8 ± 9.8 years) were collected and immediately frozen in liquid nitrogen. All human samples were stored at − 80 °C until the complete collection of the study cohort.

Demographic characteristics of the study groups are presented in Supplementary Table 1. Following dissection by a specialist forensic medicine physician, tissue aliquots were prepared from the hippocampus, putamen, and caudate nucleus of both groups.

The study was conducted according to the principles of the Declaration of Helsinki and its subsequent revisions. All experiments involving post-mortem tissue were performed in accordance with ethical approval obtained from the Istanbul Medipol University Clinical Research Ethics Committee (22.08.2014/66291034–41).

### Sample Preparation

To minimize technical variability and batch effects in this study, all brain samples from heroin users and control subjects were collected using identical procedures, immediately frozen in liquid nitrogen, and stored at − 80 °C until analysis. After the complete sample set was assembled, they were thawed simultaneously and subjected to identical pre-analytical procedures under the same experimental conditions. For sample preparation, sequential extraction, denaturation, reduction, alkylation, and trypsinization steps were performed. Briefly, the tissues were transferred into 50 mM ammonium bicarbonate (ABC) buffer (Fluka, Product FL40867-50G) supplemented with a 1X HALT protease and phosphatase inhibitor cocktail (Thermo Scientific, Rockford, IL, USA) and the tissues were homogenized using stainless steel beads. A protein extraction buffer containing 2% sodium dodecyl sulfate (SDS, Sigma-Aldrich, St. Louis, MO, USA), 100 mM Tris–HCl (pH 7.8), and 50 mM dithiothreitol (DTT, Sigma-Aldrich, St. Louis, MO, USA) was added to the homogenate at a 1:1 (v/v) ratio. The samples were then heated at 95 °C for 10 min to denature the proteins. The resulting extracts were centrifuged at 14,000 × g for 15 min to remove cellular debris. Subsequently, total protein concentrations were measured, and equal amounts from each sample were used for on-membrane digestion [19]. Samples were initially treated with 8 M urea (100 mM Tris/HCl, pH 8.5), and alkylation was subsequently performed with 40 mM iodoacetamide (Sigma-Aldrich, St. Louis, MO, USA) prepared in urea at the same molarity. The samples were incubated at room temperature in the dark.

Prior to initiating the trypsinization step, the samples were washed with ABC buffer to adjust the pH to a level suitable for enzymatic activity. Lyophilized trypsin (Pierce, Thermo Fisher Scientific, Rockford, IL, USA) was reconstituted in 50 mM acetic acid and subsequently diluted with 50 mM ABC to achieve a final pH of 7.5–8.5. The trypsin solution was added to each sample at a 1:25 ratio and incubated overnight at 37 °C. Following digestion, the samples were lyophilized and stored at −20 °C until further analysis.

### Mass Spectrometry and Data Analysis

For proteomic analysis, a Q-Exactive Plus mass spectrometer (Thermo Fisher Scientific, Waltham, USA) equipped with an electrospray ionization (ESI) source and coupled to an Ultimate 3000 liquid chromatograph was used. Lyophilized peptides were resuspended in 0.1% formic acid and loaded onto an analytical column (2 μm particle size × 75 μm inner diameter, Acclaim PepMap 100 C18, Thermo Fisher Scientific, Waltham, USA) using a linear gradient of 5–30% mobile phase B (0.1% formic acid in 95% acetonitrile, Merck, Darmstadt, Germany). Mass spectrometry (MS) and tandem MS (MS/MS) spectra of peptides introduced via ESI were acquired. Liquid chromatography-tandem mass spectrometry (LC–MS/MS) parameters were set according to a previous study [20], and the instrument was operated in data-dependent acquisition (DDA) mode with full MS scans in the 350–1800 m/z range. LC–MS/MS analyses were initiated immediately following sample preparation and performed on the same instrument using identical acquisition parameters. To further reduce potential batch-related bias, the order of LC–MS/MS runs was randomized across heroin user and control samples. These measures were implemented to ensure that observed proteomic differences primarily reflect biological variation rather than technical effects.

Raw data were processed using Proteome Discoverer (PD) version 2.4, and the acquired MS/MS spectra were searched with the Sequest search engine against the human protein database (207,933 sequences; downloaded from UniProt in November 2023). For protein identification, a total of 36 MS datasets per region and group were evaluated using the label-free method. After identification, the STRING database (https://string-db.org/) was used for gene ontology (GO) analysis and protein–protein interaction (PPI) network construction to identify the functional relationships among the significantly altered proteins and to uncover potential hub proteins involved in heroin-induced molecular dysregulation. The resulting interaction networks were visualized using Cytoscape v3.10.1.

### Statistical Analysis

P-values for group comparisons were calculated using the PD platform based on paired t-tests. Proteins were considered differentially expressed (DEPs) if they exhibited at least a twofold change in abundance (log2-fold change ≥ 1, or ≤ −1) with a p value < 0.05. Following label-free quantification, only proteins identified by two or more tryptic peptides and with a protein-level false discovery rate (FDR) ≤ 0.01 were included in further analyses.

## Results

### Global Proteome Profile

**Fig. 1:**
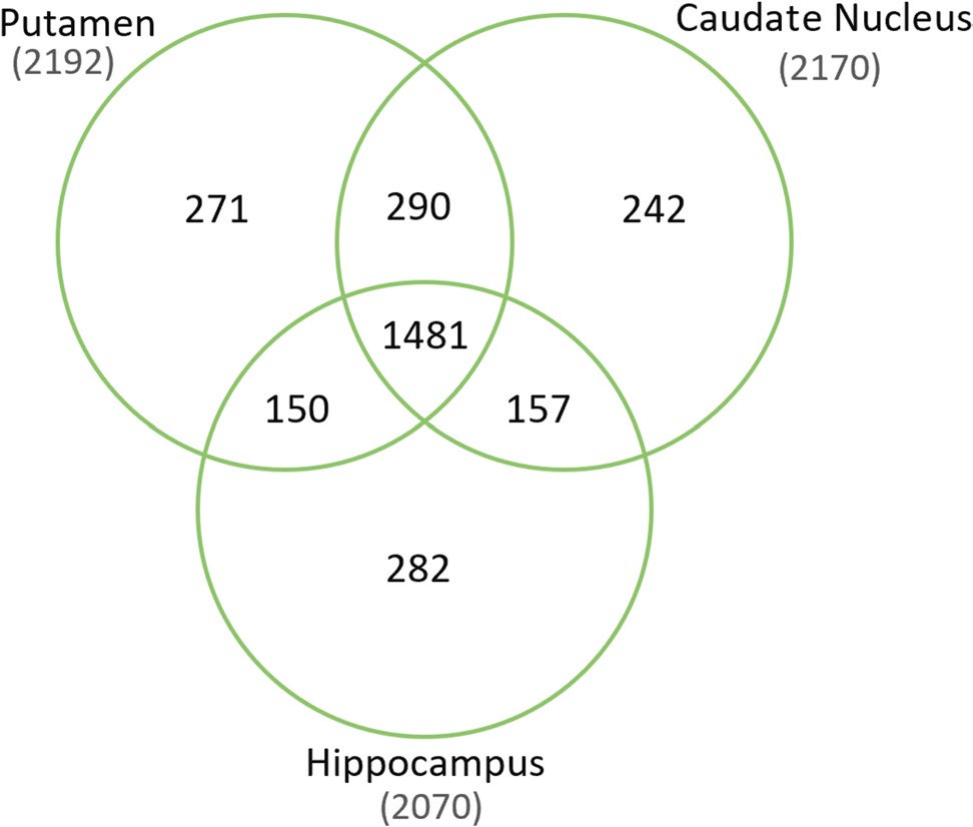
Comparison of identified protein numbers for hippocampus, putamen, and caudate nucleus. Proteins identified with high confidence, < 1% FDR

In the current study, a total of 2873 brain proteins were identified (FDR < 1%) across the hippocampus, putamen, and caudate nucleus. More than 50% of these proteins were found to be common to all three regions. The number of proteins identified in each region, as well as the shared proteins, is presented in Fig. 1.

### Differential Protein Expression and PPI Network Analysis

**Fig. 2:**
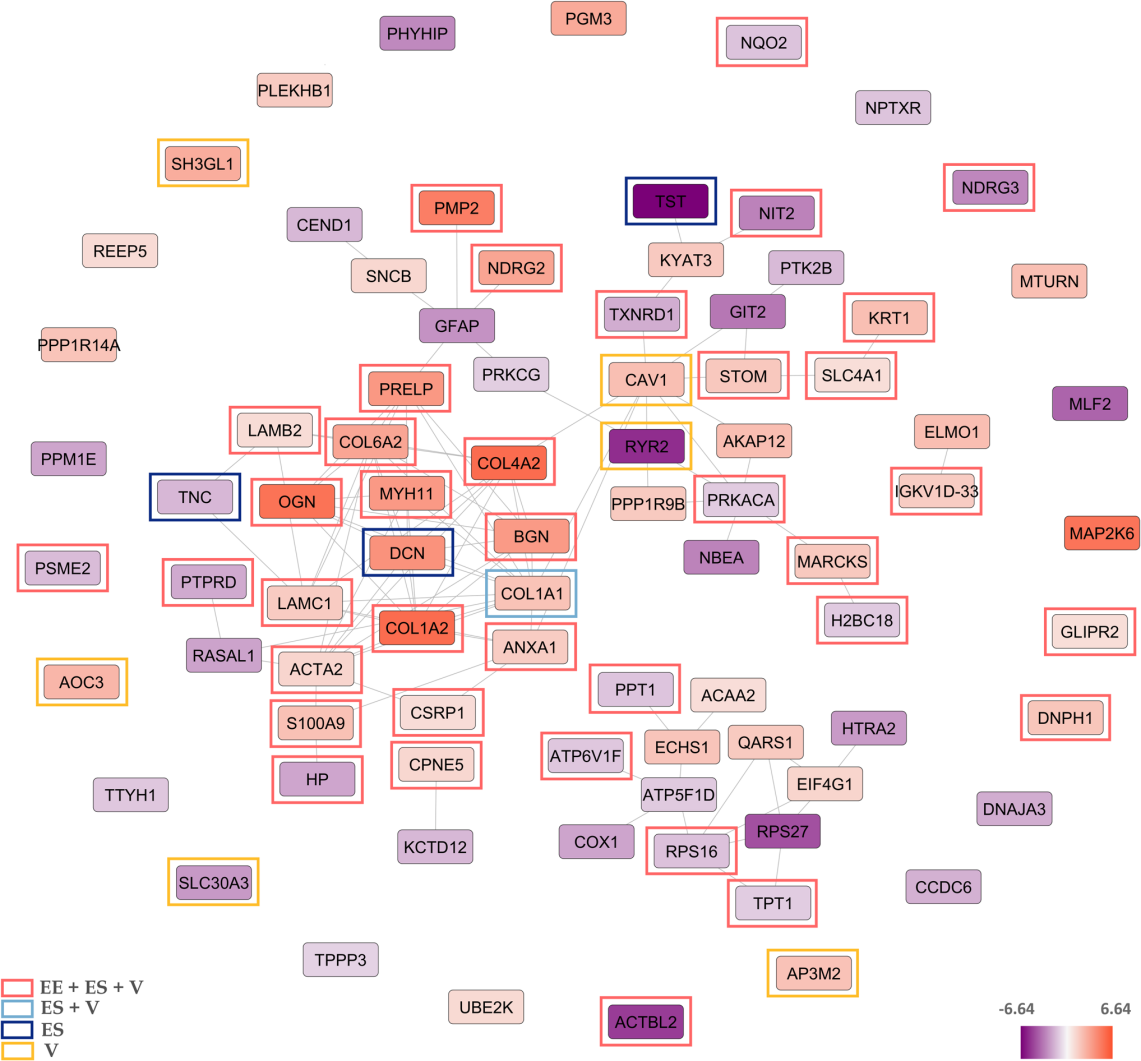
Protein–protein interaction network of DEPs in hippocampus. The network formed with differentially expressed proteins in heroin use compared to the control group. Amino acid sequences of 87 proteins were uploaded to the STRING database to build the interaction network. Network stats for hippocampus; average node degree: 2.48, avg. local clustering coefficient: 0.428, PPI enrichment p-value: < 2.69e-09. Color depth of the nodes represents expression differences. Color-coded frames indicate proteins belonging to significantly enriched cellular component categories. Abbreviations: EE, extracellular exosome; ES, extracellular space; V, Vesicle

Label-free protein quantification analysis revealed proteins that exhibited at least a twofold change in expression between the heroin users and control groups in each brain region. In the heroin user group, the expression of 47 hippocampal proteins out of the total 87 proteins significantly increased and the expression of 40 hippocampal proteins decreased compared to the control group (Supplementary Table 2). To further characterize the proteomic alterations in the hippocampus of the heroin user group, the differentially expressed proteins (DEPs) identified in this region were analysed using the STRING database to construct a PPI network allowing determination of how these proteins interact within biological pathways. To better visualize, we displayed the proteins that are enriched in categories associated with Extracellular Exosomes (EE), the Extracellular Space (ES), and Vesicle-related processes (VS) in the figures according to the Supplementary Table 3. All DEPs identified in the hippocampus (n = 87) were mapped, forming a significantly enriched interaction network (PPI enrichment p < 2.69e⁻^9^), indicating that the observed protein changes were not random but represented biologically meaningful connectivity. The network showed an average node degree of 2.48 and an average local clustering coefficient of 0.428, suggesting a functionally relevant interaction density among these proteins (Fig. 2). Several extracellular matrix–related proteins such as COL1A1, COL1A2, COL4A2, BGN, DCN, MYH11, PMP2, MAP2K6, and OGN were strongly upregulated and formed a highly connected cluster, indicating a coordinated alteration in structural and matrix-associated pathways. Conversely, metabolic and mitochondrial proteins such as COX1, ATP5F1D, TST, RYR2 and RPS27 were predominantly downregulated, consistent with potential energy metabolism dysfunction in the hippocampus. Central regulatory nodes such as CAV1, RYR2, PRKCG, and TXNRD1 displayed multiple interactions within the network, suggesting they may act as intermediates linking signaling, redox regulation, and cytoskeletal remodeling (Fig. 2).

**Fig. 3:**
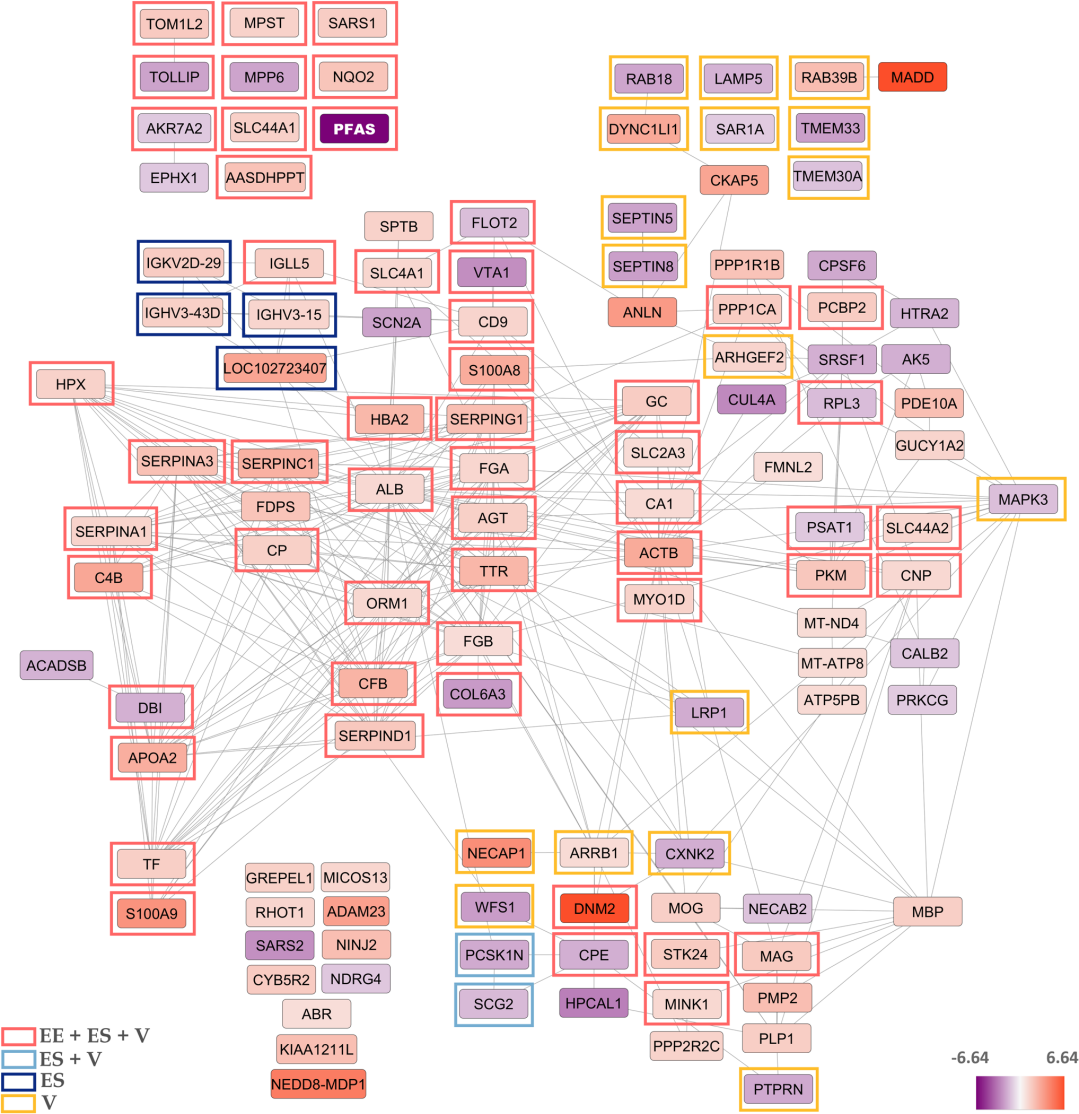
Protein–protein interaction network of DEPs in putamen. The network formed with differentially expressed proteins in heroin use compared to the control group. Amino acid sequences of 121 proteins were uploaded to the STRING database to build the interaction network. Network stats for putamen; average node degree: 5.4, avg. local clustering coefficient: 0.457, PPI enrichment p-value: < 1.0e-16. Color depth of the nodes represents expression differences. Color-coded frames indicate proteins belonging to significantly enriched cellular component categories. Abbreviations: EE, extracellular exosome; ES, extracellular space; V, Vesicle

Similarly, in the putamen of the heroin user group, all DEPs identified in comparison with the control group (n = 121) were mapped into a PPI network using the STRING database (Fig. 3, Supplementary Table 2). The resulting network displayed a high level of interconnectedness, with an average node degree of 5.4 and an average local clustering coefficient of 0.457, indicating dense functional associations among the altered proteins. The enrichment was highly significant (PPI enrichment p < 1.0e⁻^16^), demonstrating that the observed interactions were unlikely to occur by chance. Among these proteins, the expression of 83 proteins increased while the expression of 38 proteins decreased. The most dramatic increases were observed in the expressions of DNM2 and MADD proteins. In addition, a large group of acute-phase, immune-related, and complement pathway proteins including SERPINs, CFB, C4B, HPX, ALB, FGA, FGB, and S100A8/A9, showed prominent connectivity, suggesting an enhanced inflammatory and immune response in the putamen of the heroin user group. Another highly connected cluster consisted of myelin- and axon-related proteins such as MBP, MOG, MAG, PMP2, PLP1, CNP, and cytoskeletal regulators (ACTB, FMNL2, MYO1D), indicating potential alterations in myelination and neuronal structural integrity. Additionally, several signaling and vesicle-associated proteins, including DNM2, NECAP1, ARRB1, PPP2R2C, SCG2, and HPCAL1, formed a distinct interaction hub, pointing to disruptions in endocytosis, synaptic transmission, and intracellular communication. Mitochondrial proteins (e.g., MT-ND4, MT-ATP8, ATP5PB) were predominantly downregulated, consistent with a possible impairment of oxidative phosphorylation and energy metabolism in the putamen (Fig. 3).

**Fig. 4:**
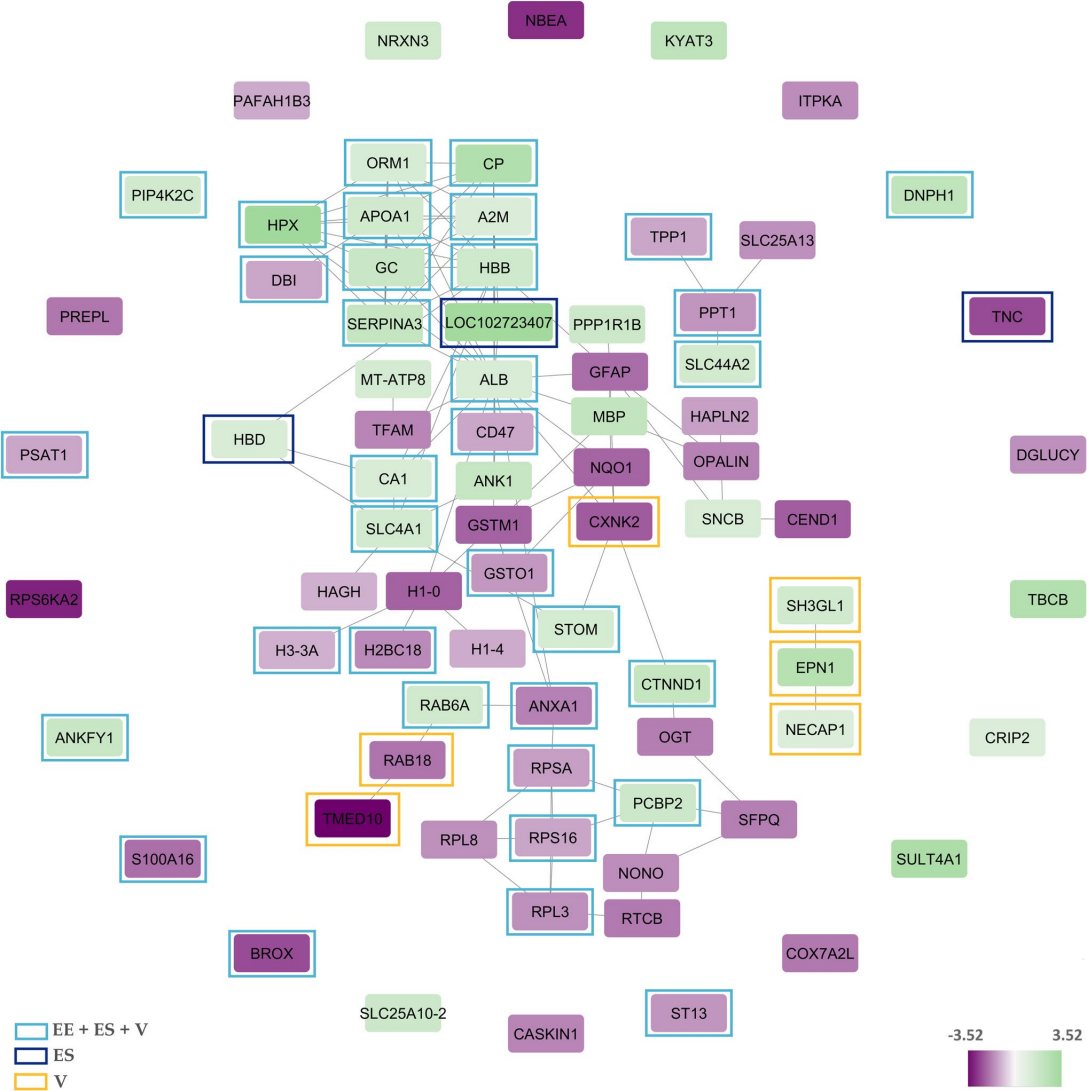
Protein–protein interaction network of DEPs in caudate nucleus. The network is formed with differentially expressed proteins in heroin use compared to the control group. Amino acid sequences of 87 proteins were uploaded to STRING database to build the interaction network. Network stats for the hippocampus; average node degree: 2.72, avg. local clustering coefficient: 0.439, PPI enrichment p-value: 1.87e-10. Color depth of the nodes represents expression differences. Color-coded frames indicate proteins belonging to significantly enriched cellular component categories. Abbreviations: EE, extracellular exosome; ES, extracellular space; V, Vesicle

In the caudate nucleus, compared to the control group, significant expression changes were observed in 80 proteins of heroin user group, of which 23 were increased and 43 decreased (Supplementary Table 2). All DEPs identified in this comparison were used as input for STRING to construct the PPI network. The resulting network exhibited significant enrichment, with an average node degree of 2.72 and a clustering coefficient of 0.439 (PPI enrichment p = 1.87e⁻^10^). A broad group of transport and plasma-related proteins, including ALB, ORM1, APOA1, CP, A2M, HBB, and HBD, formed a central hub, suggesting disturbances in metabolic transport and plasma-derived protein infiltration in the caudate region. Another coherent cluster consisted of glial and myelin-associated proteins such as GFAP, MBP, OPALIN, HAPLN2, and SNCB, implying heroin-related effects on glial activation and myelin structure. Oxidative stress-related and detoxification enzymes, including NQO1, GSTM1, GSTO1, HAGH, and CA1, were predominantly downregulated, pointing toward impaired redox homeostasis. Additional clusters included proteins involved in ribosomal function and RNA processing (e.g., RPL3, RPL8, RPS16, PCBP2, NONO, RTCB) and vesicle trafficking and signalling proteins such as RAB18, RAB6A, TMED10, and ANK1. In addition, the expressions of RPS6KA2 and NBEA proteins were dramatically decreased (Fig. 4). These changes collectively suggest disruptions in protein synthesis, intracellular transport, and synaptic signalling in the caudate nucleus.

In addition, five proteins (S100A9, HTRA2, PMP2, SLC4A1, IGHG4) were common to the hippocampus and putamen, while eighteen proteins (RAB18, RPL3, HPX, PPP1R1B, ALB, PCBP2, CA1, PSAT1, SLC44A2, ORM1, ATP8, NECAP1, GC, LOC102723407, DBI, CXNK2, SLC4A1, IGHG4) were common to the hippocampus and caudate nucleus. In addition, fifteen proteins (SNCB, TNC, CEND1, SNC73, SH3GL1, PPT1, KYAT3, RPS16, STOM, DNPH1, NBEA, H2BC18, ANXA1, SLC4A1, IGHG4) were common to the putamen and caudate nucleus.

**Fig. 5:**
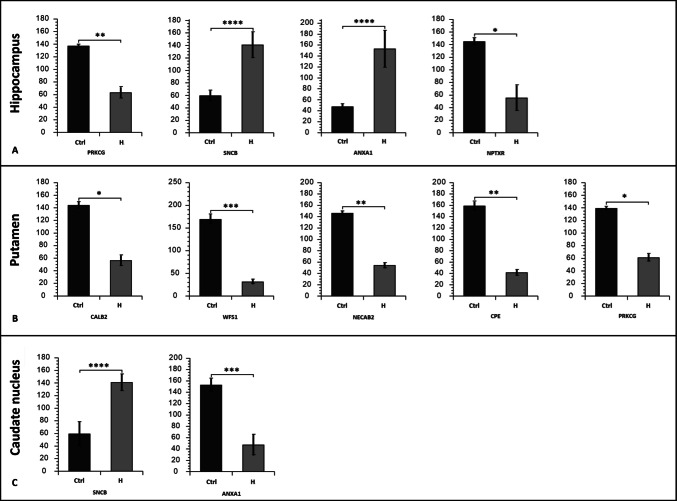
The bar graph of highlighted DEPs related to neurodegeneration. Bar graphs showing normalized protein abundance values (arbitrary units, a.u.) of differentially expressed proteins (DEPs) associated with neurodegeneration across different brain regions. For each protein, expression levels were compared between the heroin use (H) and control (Ctrl) groups. Proteins identified in the hippocampus are shown in panel A, those in the putamen in panel B, and those in the caudate nucleus in panel C. Differential expression was determined by label-free quantification (fold change > 2 with statistical significance and at least two peptides). *, *p* < 0.05, **, *p* < 0.01; ***, 0.001; ****, *p* < 0.0001

Given the well-documented impact of chronic opioid exposure on brain function, we examined whether proteins previously associated with neurodegenerative processes exhibited altered expression patterns across brain regions. Among the proteins showing altered expression in our dataset were several previously implicated in neurodegenerative processes, indicating a possible association between heroin exposure and pathways relevant to neurodegeneration. In the hippocampus, the expression of PRKCG (log2FC: −1.12, *p* < 0.01) and Neuronal pentraxin receptor (NPTXR; log2FC: −1.39, *p* < 0.05) decreased, while the expression of beta synuclein (SNCB; log2FC: 1.25, p < 0.0001) and annexin A1 (ANXA1; log2FC: 1.7, *p* < 0.0001) increased (Fig. 5A). In the caudate nucleus, a 2.39-fold increase in beta synuclein levels (log2FC: 1.26, *p* < 0.0001) was observed. Interestingly, although ANXA1 expression was elevated in the hippocampus, it was decreased by 0.31-fold in the caudate nucleus (log2FC: −1.69, p < 0.001) (Fig. 5B). In the putamen, calretinin (CALB2; log2FC: −1.36, *p* < 0.05), wolframin (WFS1; log2FC: −2.45, p < 0.001), N-terminal EF-hand calcium-binding protein 2 (NECAB2; log2FC: −1.43, *p* < 0.01), Carboxypeptidase E (CPE; log2FC: −1.94, *p* < 0.01), and Protein Kinase C (PRKCG; log2FC: −1.19, *p* < 0.05) showed significant alterations (Fig. 5C). Additionally, alterations were detected in several proteins and kinases exhibiting catalytic activity, some of which are known to be associated with neurodegeneration (Supplementary Table 3).

### Functional Enrichment Analysis: GO Terms and Pathways

Functional enrichment analysis, including GO categories (biological process, molecular function, and cellular component) and pathway analysis, was performed using the STRING database based on the same set of DEPs used for PPI network construction. The top enriched pathways directly associated with the core set of DEPs are presented in Table 1 and the complete list of all enriched pathways generated by the analysis pipeline is presented in Supplementary Table 3. Pathway enrichment analysis revealed that proteins dysregulated in the hippocampus were significantly associated with extracellular matrix (ECM) proteoglycans, ECM organization, signaling by receptor tyrosine kinases, and focal adhesion pathways. In the putamen, altered proteins were mainly linked to homeostasis, platelet activation and signaling, protein phosphorylation, and IGF regulation. In the caudate nucleus, dysregulated proteins were significantly enriched in pathways related to metabolism, cellular response to stimuli and stress, and vesicle-mediated transport (Table 1).

**Table 1:** Functional enrichment pathway by STRING

	**Hippocampus**			
	**# ID**	**Description**	**Observed Count**	**FDR**
REACTOME Pathway	HSA-3000178	ECM proteoglycans	9	2.09e-07
	HSA-3000171	Non-integrin membrane-ECM interactions	6	0.00038
	HSA-9006934	Signaling by Receptor Tyrosine Kinases	13	0.00038
	HSA-1474244	Extracellular matrix organization	10	0.00044
	HSA-216083	Integrin cell surface interactions	6	0.0011
Wiki Pathway	WP2911	miRNA targets in ECM and membrane receptors	5	7.17e-05
	WP306	Focal adhesion	9	8.14e-05
	WP5055	Burn wound healing	6	0.0015
	WP5036	Angiotensin II receptor type 1 pathway	4	0.0019
	WP453	Inflammatory response pathway	4	0.0020
	**Putamen**			
	**# ID**	**Description**	**Observed Count**	**FDR**
REACTOME Pathway	HSA-8957275	Post-translational protein phosphorylation	10	4.18e-06
	HSA-381426	Regulation of Insulin-like Growth Factor (IGF) transport and uptake by Insulin-like Growth Factor Binding Proteins (IGFBPs)	10	7.85e-06
	HSA-76005	Response to elevated platelet cytosolic Ca2 +	10	8.57e-06
	HSA-109582	Hemostasis	18	1.55e-05
	HSA-76002	Platelet activation, signaling and aggregation	12	3.42e-05
Wiki Pathway	WP558	Complement and coagulation cascades	7	8.55e-05
	WP2276	Glial cell differentiation	4	0.00014
	WP4304	Oligodendrocyte specification and differentiation, leading to myelin components for CNS	5	0.00048
	WP176	Folate metabolism	5	0.0116
	WP5115	Network map of SARS-CoV-2 signaling pathway	8	0.0116
	**Caudate Nucleus**			
	**# ID**	**Description**	**Observed Count**	**FDR**
REACTOME Pathway	HSA-1430728	Metabolism	25	0.00065
	HSA-2168880	Scavenging of heme from plasma	4	0.00065
	HSA-8953897	Cellular responses to stimuli	15	0.00065
	HSA-2262752	Cellular responses to stress	14	0.00082
	HSA-5653656	Vesicle-mediated transport	13	0.0011
Wiki Pathway	WP1533	Vitamin B12 metabolism	4	0.0259
	WP2431	Spinal cord injury	5	0.0259
	WP3888	VEGFA-VEGFR2 signaling	9	0.0259
	WP477	Cytoplasmic ribosomal proteins	5	0.0259
	WP176	Folate metabolism	4	0.0278

**Fig. 6:**
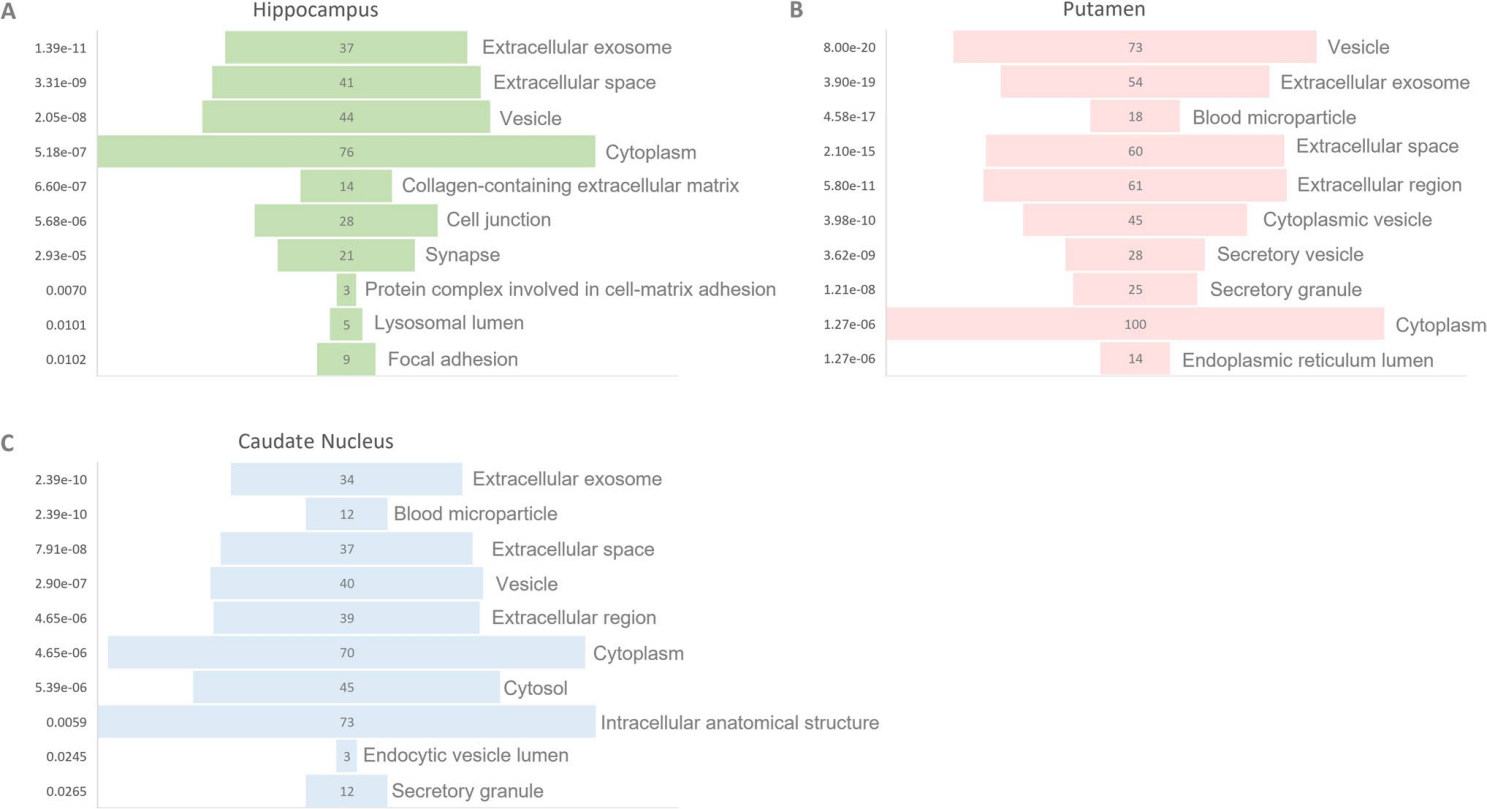
GO analysis of DEPs. GO terms based on cellular component. The graph shows the top 10 enriched categories in the hippocampus (**A**), putamen (**B**), and caudate nucleus (**C**). The y-axis indicates FDR. Biological process and molecular function annotations are also given in Supplementary Table 3

Across all three regions, the most enriched cellular components were extracellular exosome, extracellular space, and vesicles. In the hippocampus, although most DEPs were localized to the cytoplasm, several were linked to key structural components such as the collagen-containing extracellular matrix, cell junctions, and synapses (Fig. 6A). GO molecular function (MF) enrichment analysis indicated that proteins associated with this region were significantly enriched in the categories of structural molecule activity, ECM structural constituent, and kinase binding. Notably, a substantial proportion of the kinase-binding proteins were associated with cell differentiation (Supplementary Table 3). In the caudate nucleus, the proteins altered by heroin exposure were predominantly associated with intracellular anatomical structures as well as the cytoplasm (Fig. 6B). However, no significant enrichment was observed in the GO biological process (BP) or MF categories for this region under the applied criteria (Supplementary Table 3). In the putamen, many proteins involved in stress response were localized within these structures (Fig. 6C). The enriched MF categories included signaling, binding, small molecule binding, and inhibitory activity, whereas the top BP categories were transport, localization, and stress response (Supplementary Table 3).

## Discussion

Addictive drugs are known to cause damaging changes in various regions of the brain limbic system [21]. Numerous proteins have been identified as potential molecules involved in the molecular mechanisms underlying drug dependence using various analytical techniques [22]. In the present study, proteomic changes in three brain regions (hippocampus, putamen, and caudate nucleus) were investigated to identify the proteins associated with the molecular mechanisms of heroin use in humans. In total, MS-based analyses enabled the high-confidence identification of 2873 proteins. Compared to the control group, significant expression changes were detected in proteins primarily located in the cytoplasm of central nervous system cells in the heroin user group. Several of these proteins are known to play roles in stress responses, calcium homeostasis, cytoskeletal organization, and energy metabolism. Although our proteomic analysis revealed widespread alterations across multiple functional pathways, we focused our discussion on proteins previously associated with neurodegenerative processes. This emphasis was motivated by the fact that these proteins not only showed consistent, region-specific expression changes in our dataset but also aligned with existing evidence linking chronic opioid exposure to neurodegenerative-like mechanisms.

According to the proteomic analysis of the hippocampus, nearly a 6.5-fold decrease was observed in thiosulfate sulfurtransferase (TST), a detoxifying enzyme with rhodanese activity located in the mitochondria that detoxifies cyanide using thiosulfate as a substrate [23]. Recent studies have shown that TST also has important functions in hydrogen sulfide metabolism, antioxidant defense, and several protective biological processes including mitochondrial homeostasis [23]. Therefore, the dramatic decrease in TST expression due to heroin addiction may result in an increase in oxidative stress which is highly detrimental to neuronal activity. It is known that TST functions in two thiol-dependent antioxidant systems, the thioredoxin system and the glutathione (GSH) system [24]. By donating sulfur within these systems, TST plays an important role as a reactive oxygen species (ROS) scavenger [25]. The hippocampus, a central region for learning and memory formation, is particularly sensitive to oxidative stress [26]. Therefore, an increase in oxygen radicals in the hippocampus may predispose individuals to various pathological conditions, including neurodegenerative diseases such as Alzheimer’s disease (AD) and Parkinson’s disease (PD). Previous studies have also shown that the absence or reduction of TST activity in the brain results in pathophysiological conditions associated with oxidative stress-related dysfunction [23].

The decrease in TST expression was accompanied by a reduction in the redox-sensitive ryanodine receptor 2 (RYR2) which interacts with TST in the protein–protein interaction map (Fig. 2). In hippocampal neurons, RYR2 plays an essential role in synaptic plasticity, learning and memory formation, and memory consolidation by releasing Ca^2+^ from the endoplasmic reticulum [27]. Because RyR-mediated Ca^2+^ release is required for hippocampal learning and memory processes, a dramatic heroin-dependent decrease in RYR2 expression may contribute to memory impairments such as dementias [28]. Previous studies have also shown that dysregulated RyR-mediated Ca^2+^ release due to decreased RYR2 expression contributes to aging and neurodegenerative diseases such as AD [29, 30]. In the hippocampus of heroin users, actin beta like 2 (ACTBL2) was also significantly decreased compared to controls. ACTBL2 is an actin isoform structurally similar to beta-actin [31]. The significant decrease in ACTBL2 may be related to loss of neuronal structures because actin filaments maintain cell morphology and motility. Previous studies have demonstrated that ACTBL2 levels are significantly increased in highly proliferative cancer cells that require cellular remodeling [31–33]. Since memory formation relies on synaptic plasticity, and chronic exposure to drugs of abuse induces persistent alterations in synaptic structure and memory formation [34], heroin-related reductions in ACTBL2 may disrupt actin-dependent dendritic and spine remodeling, contributing to neuronal loss and neurodegeneration in the hippocampus [35]. Another significantly downregulated protein in hippocampal tissue from heroin users was the 40S ribosomal protein S27 (RPS27), which is typically overexpressed in proliferative tissues and cancer cells [36]. Consistent with this finding, a previous study reported that heroin abuse significantly decreased another ribosomal protein, ribosomal protein S14, in human astrocytes [37]. Thus, the pronounced significant downregulation of RPS27 observed in the present study may be related to a reduced protein synthesis capacity resulting in neuronal loss or dysfunction. Supporting our results, recent evidence suggests that reduced RPS27 levels may serve as a characteristic biomarker for AD screening [38].

On the other hand, heroin use significantly increased the expression of collagen type IV alpha chains (COL4A2). Members of the collagen superfamily are crucial for neuronal maturation, neural circuit formation, axon guidance, and synaptogenesis. Because collagen in the nervous system is highly dynamic and contains multiple binding sites for matrix proteins, receptors, and ligands such as pro-inflammatory signals, alterations in its deposition can modify cellular responses and contribute to the limited regenerative capacity of neural tissue [39]. While collagen I plays important roles in neuronal maturation, neural circuit formation, axon guidance, and synaptogenesis, collagen IV forms a major structural scaffold to which other ECM-associated proteins bind and interact [40, 41]. In previous studies, increased collagen IV deposition in the microvessels of AD brains has been reported. Additionally, the binding domains of type I collagen for amyloid precursor protein have been shown to contribute to monocyte recruitment during disease states [42, 43]. Similarly, the expression of osteoglycin (OGN), a small keratan sulfate proteoglycan, was elevated in post-mortem hippocampal tissue from heroin users. In addition to its role in bone formation, OGN has been implicated in vascular pathologies through its effects on cellular proliferation [44, 45]. Therefore, heroin-related upregulation of these collagens and OGN may disrupt extracellular matrix organization, thereby impairing the regenerative capacity of hippocampal tissue and contributing to increased neurodegeneration.

In addition to extracellular matrix proteins, the expression of myelin P2 protein (PMP2), which stabilizes myelin membranes, was increased in the hippocampal tissue of heroin users. Molecular studies have shown that PMP2 facilitates the endocytosis-independent transport of sphingomyelin from the outer to the inner leaflet of the plasma membrane. In the overexpression of PMP2, the distribution of sphingomyelin across the membrane becomes disrupted, leading to a relative decrease in the outer leaflet where its concentration is normally higher [46]. Such an imbalance may alter sphingomyelin-associated signal transduction pathways and represent an early step in heroin-induced disruption of neuronal signaling. Supporting this notion, the expression of MAP kinase 6 (MAP2K6), a key component of sphingomyelin-related signaling pathways, was also increased in the hippocampus of heroin users [47]. Activation of this kinase may function in several cellular processes including stress-related cellular responses and apoptosis [48]. This suggests that a heroin-related decrease in extracellular sphingomyelin, together with increased MAP2K6 activity, may increase stress-induced apoptotic cell death in hippocampal neurons. Moreover, elevated MAP2K6 levels are known to impair synaptic plasticity and memory by affecting metabotropic glutamate receptor-dependent long-term depression at CA3–CA1 synapses [49].

In hippocampal neurons, heroin use may increase oxidative stress, which can disrupt synaptic plasticity by altering extracellular matrix organization and impairing key signal transduction pathways. Such disturbances may contribute to neurodegenerative processes observed in disorders like AD. Our current data are consistent with previous studies showing that long-term exposure to addictive drugs can lead to adverse molecular alterations including synaptic dysfunction, impaired neurogenesis, neuronal loss, and disrupted neuroplasticity that deteriorate hippocampal function and resemble pathological features observed in neurodegenerative diseases including AD and PD [11, 50–53].

In AD, beta-amyloid accumulation and the reduced capacity to buffer rising intracellular calcium against decreasing calcium binding protein levels can lead to neurotoxicity and trigger apoptotic neuronal death [54]. In our study, we similarly observed a significant reduction in several neuroprotective calcium-binding proteins such as calretinin, wolframin, and NECAB2. According to preliminary findings, calretinin-positive neurons are more resistant to beta-amyloid toxicity and other neurotoxic insults [55]. Findings to date have also shown decreased levels of calretinin in AD brains [56–58]. Wolframin, another calcium-binding protein, contributes to tau pathology and neurodegeneration through mechanisms involving chronic endoplasmic reticulum stress [59, 60]. Therefore, the decreased expression of calretinin, wolframin, and NECAB2 in the hippocampus may reflect heroin-induced disturbances in calcium homeostasis. It is well established that long-term increases in intracellular calcium, resulting from impaired calcium regulation, exert neurotoxic effects and can initiate apoptotic pathways. Moreover, the increased hippocampal expression of ANXA1, which plays a stimulatory role in the microglia-mediated phagocytic clearance of apoptotic neurons, further supports the presence of heroin-induced neurodegeneration [61, 62].

In general, it has been reported that chronic exposure to addictive drugs, including heroin, causes decreases in protein kinase C (PKC) α and β levels, which are associated with neurodegeneration, neuronal death, and learning and memory impairments [63]. Interestingly, LC–MS/MS analysis showed that PKC expression was decreased in the hippocampus of the heroin user group. Another important finding of this study was the increased expression of beta-synuclein in the hippocampus. There is substantial evidence demonstrating an increase in beta-synuclein, an important marker of synaptic degeneration, in the cerebrospinal fluid (CSF) of patients with AD [64, 65]. In addition, NPTXR, another protein linked to synaptic integrity, was found to be reduced in the hippocampus of heroin users. Previous studies conducted in CSF have reported that decreased NPTXR levels may serve as a promising biomarker for predicting AD progression and severity [65–67].

In the literature, the putamen has mostly been reported to show no major protein alterations due to drug and alcohol use, whereas heroin abusers have exhibited overexpression of certain receptor types [68–71]. In the putamen of our heroin user group, dynamin-2 (DNM2), a member of the GTP-binding protein family, was significantly increased. Dynamins are associated with microtubules and function in cellular processes such as endocytosis and cell motility [701]. Previously, an increase in DNM2 expression has been observed with increasing age [72]. In addition, DNM2 plays a role in amyloid-beta internalization through endocytosis [73]. Another heroin-upregulated protein in the putamen was MAP kinase-activating death domain protein (MADD). Overexpression of MADD activates the mitogen-activated protein (MAP) kinase and extracellular signal-regulated kinase (ERK), which induces phosphorylation of cytosolic phospholipase A2 and leads to neuronal cell death [74]. In addition to these proteins, the significant decrease in carboxypeptidase E (CPE) expression in the putamen of the heroin user group may increase neurodegeneration because CPE levels decrease with age and contribute to reduced neurogenesis in older individuals [75]. The decrease in PKC expression in the putamen further supports findings related to neurodegeneration. Decreased expression levels of calretinin, wolframin, and NECAB2 were also observed in the putamen of the heroin user group, indicating heroin-induced calcium imbalance similar to that seen in the hippocampus. The caudate nucleus is another brain region showing functional alterations following drug administration. For instance, acute heroin administration increased activity in the caudate-putamen region of drug-naïve rats [76]. In our study, a significant decrease in ribosomal protein S6 kinase alpha-2 (RPS6KA2), which is implicated in controlling cell growth and differentiation, was observed in post-mortem tissue from heroin users [77]. In the literature, reduced RPS6KA2 levels have been reported in the caudate of post-mortem Huntington’s disease patients [78]. Additionally, low RPS6KA2 levels have been shown to reduce acute morphine analgesia through a specific mu-opioid receptor-mediated response [79]. Another significantly decreased protein in the caudate nucleus of the heroin user group was transmembrane emp24 domain-containing protein 10 (TMED10). By regulating the gamma-secretase activity of the secretase complex, TMED10 plays a role in the pathophysiology of AD [80]. Patients with AD have been shown to have lower levels of total TMED10 in the frontal cortex and hippocampus, where downregulation of TMED10 induces autophagy by activating autophagy-related proteins [81, 82]. Reduced TMED10 expression has also been reported in patients with schizophrenia, in whom abnormal regulation of GPI-anchored protein trafficking from the endoplasmic reticulum has been observed [83, 84]. In addition, neurobeachin (NBEA), a protein implicated in membrane protein trafficking, was also decreased in the heroin user group. As a component of neuronal synapses, reduced NBEA expression suggests that heroin may disrupt synapse formation by interfering with synaptic membrane trafficking [85]. In a previous study, knockdown of NBEA resulted in a significant increase in ethanol preference, indicating its role in drug-seeking behavior [86]. On the other hand, upregulation of hemopexin (HPX) was another heroin-induced molecular alteration observed in the caudate nucleus. While the primary function of HPX is to bind heme and reduce heme toxicity, increased HPX levels result in memory impairments in mice [87]. Because heme/iron dyshomeostasis is an early and crucial event in AD pathophysiology, the heroin-related increases in HPX may also indicate neurodegeneration. Similarly, an increase in the level of beta-synuclein in the caudate nucleus, as observed in the hippocampus, further supports heroin-induced neurodegeneration.

According to the results of this LC–MS/MS–based study, heroin affects the human hippocampus, caudate nucleus, and putamen through dysregulated proteins that are predominantly enriched in cellular structures such as extracellular exosomes, extracellular space, and vesicles. Chronic opioid exposure has been reported to influence extracellular vesicle (EV) biology, including EV-mediated neuroinflammatory signaling, synaptic remodeling, and glia–neuron communication [88]. Exosomes, in particular, play key roles in the intercellular transfer of proteins, lipids, and non-coding RNAs, and several studies have shown that opioids can alter both the content and release of EVs [88]. Therefore, the consistent enrichment of dysregulated proteins within exosome/vesicle-related categories across multiple regions may reflect opioid-induced disturbances in intercellular communication, neuroimmune activation, and trafficking pathways [89]. In addition to their role in central nervous system disorders, this pattern may also support the role of EVs in the exacerbation of substance use through heroin-dependent dysregulation of proteins in the extracellular exosome, extracellular space, and vesicles. On the other hand, heroin use was also found to predominantly disrupt transport and protein-binding mechanisms in the putamen, as well as developmental processes in the hippocampus. Indeed, the putamen plays a key role in drug-craving mechanisms, partly through the regulation of transport proteins in neuronal membranes [90]. Dysregulation of receptor signaling and ion transport mechanisms has also been reported in the putamen of patients with PD [91]. Similarly, previous studies have documented the effects of drugs of abuse on various forms of synaptic plasticity and adult neurogenesis in the hippocampus, as well as the relationship between impaired synaptic plasticity and the development of AD [92, 93]. Together, these findings further support the presence of heroin-induced neurodegenerative processes.

These heroin-induced alterations in protein expression suggest that heroin increases oxidative stress, which can disrupt synaptic plasticity in the hippocampus by altering extracellular matrix organization and key signal transduction pathways. The upregulated and downregulated proteins identified in heroin user brain tissue showed similarities to proteins with abnormal expression patterns observed in neurodegenerative diseases. Findings from the caudate nucleus and putamen further supported the presence of heroin-induced neurodegeneration. Overall, the adverse molecular changes observed in the hippocampus, putamen, and caudate nucleus due to heroin use may contribute to impaired neuronal function and synaptic plasticity, inhibition of neurogenesis and synaptic trafficking, and increased neuronal death.

This study has several limitations because it was conducted on human post-mortem brain tissue. First, post-mortem human brain studies are limited by confounding variables, including undocumented drug use history, psychiatric comorbidities, and lifestyle factors, and their cross-sectional design prevents longitudinal analysis and assessment of pre-drug neurobiological states, as observed in previous studies [69, 94]. Second, the process of obtaining the post-mortem human brain tissues was quite difficult, and this challenge significantly limited the sample size. Given the limited number of samples, the study focused on characterizing the protein profile of human brain tissues from heroin users. For this reason, validation studies of the altered proteins could not be performed using different methods in a larger sample. Additionally, since phosphoproteomic analyses could not be conducted, limited information was obtained regarding the kinases identified in the study. A further limitation of the present study is that the cohort consisted exclusively of male subjects that limit the generalizability of the findings to females. Future studies including female subjects will be essential to determine potential sex-dependent molecular alterations due to heroin use.

To summarize, the large-scale proteomic analyses presented in this study not only enhance our understanding of the molecular effects of heroin use on the human brain but also provide potential biological targets for neurodegenerative diseases, especially AD. Consequently, the altered protein expression patterns offer new clues for future research and may contribute to the development of promising therapies for the prevention or treatment of neuropathological disorders.

## Supplementary Information

Below is the link to the electronic supplementary material.

Supplementary file1 (XLSX 40 kb)

Supplementary file2 (XLSX 18 kb)

Supplementary file3 (XLSX 12 kb)

## Data Availability

The data supporting the findings of this study are subject to certain institutional and ethical restrictions. Therefore, the datasets are not publicly available but can be shared by the corresponding author upon reasonable request.
